# Supplementary figures and images for: The mitochondrial permeability transition pore activates the mitochondrial unfolded protein response and promotes aging
Source: eLife. 2021 Sep 1;10:e63453. doi: 10.7554/eLife.63453 (PMC8410078; doi:10.7554/eLife.63453)

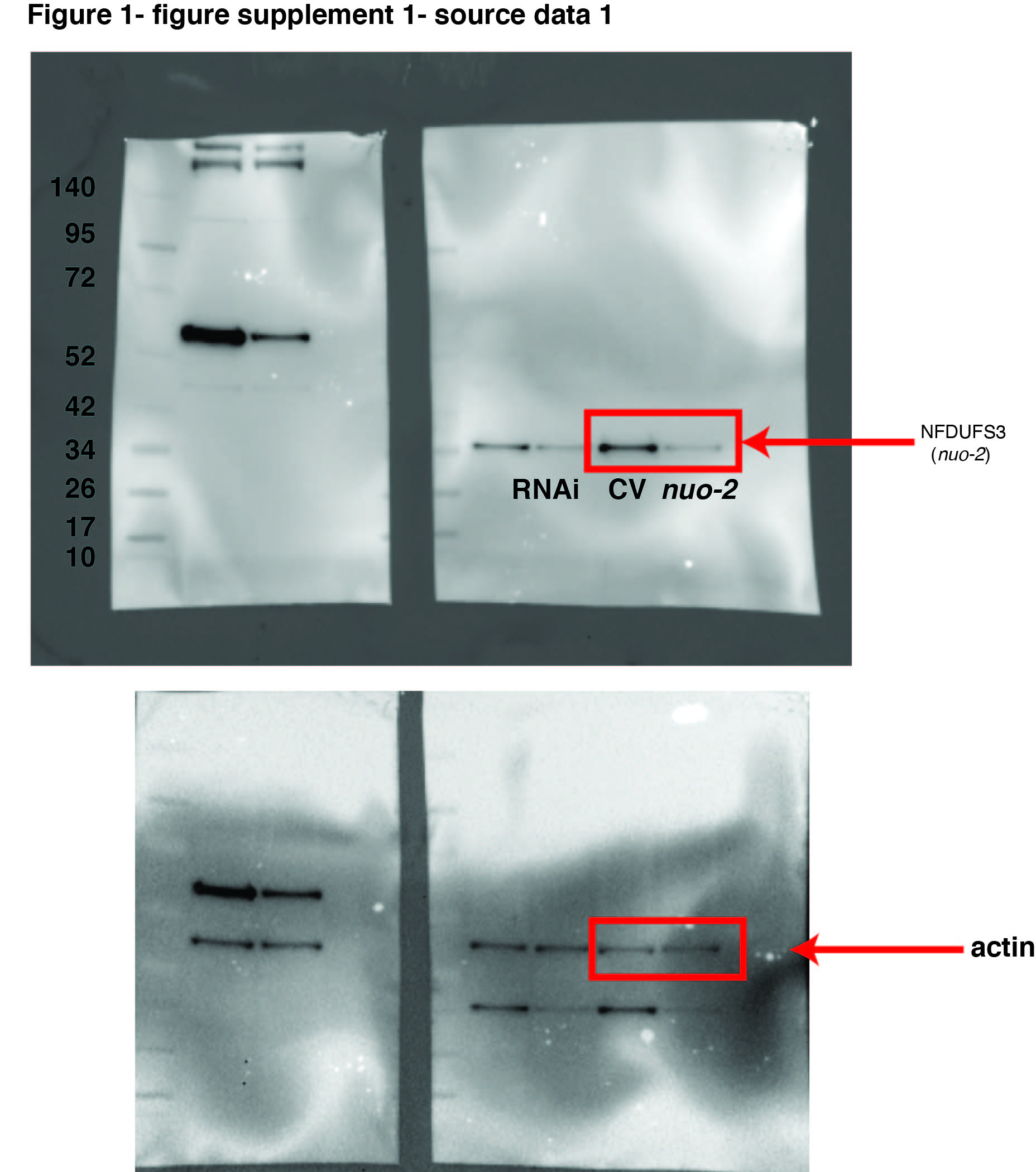

Supplement: Figure 1—figure supplement 1—source data 1. [file elife-63453-fig1-figsupp1-data1.jpg.zip › Figure 1- figure supplement 1- source data 1.jpg]

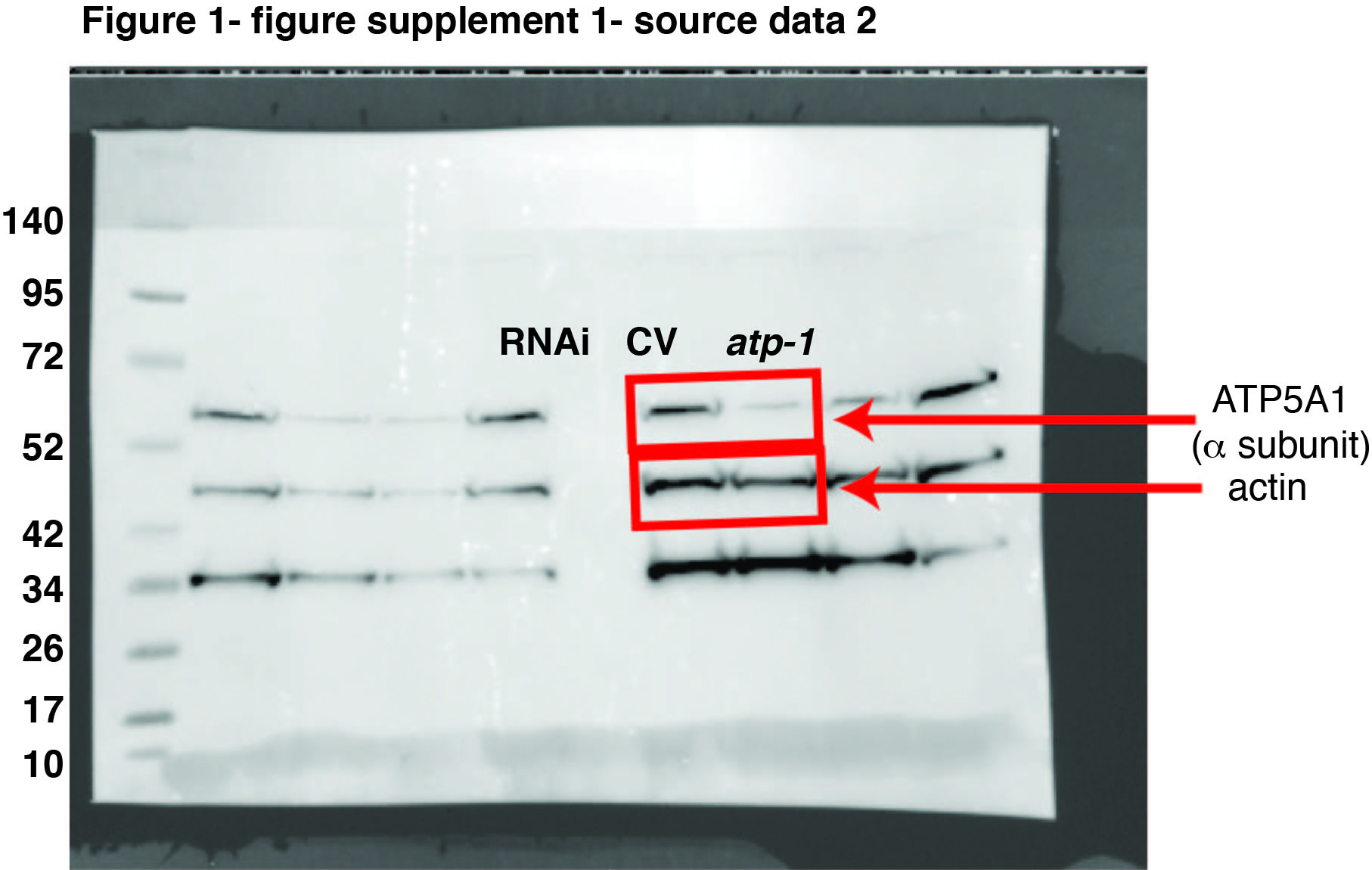

Supplement: Figure 1—figure supplement 1—source data 2. [file elife-63453-fig1-figsupp1-data2.jpg.zip › Figure 1- figure supplement 1- source data 2.jpg]

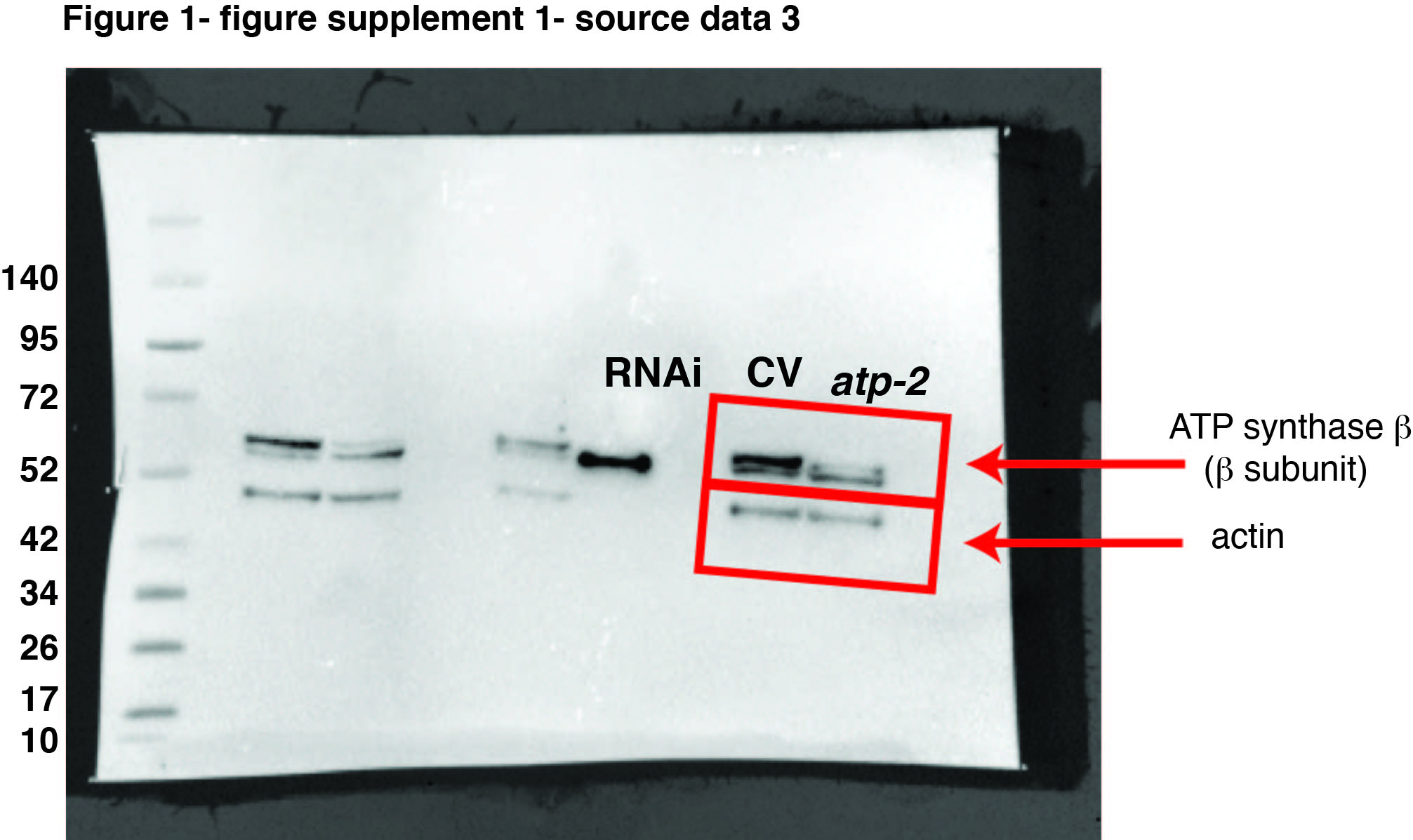

Supplement: Figure 1—figure supplement 1—source data 3. [file elife-63453-fig1-figsupp1-data3.jpg.zip › Figure 1- figure supplement 1- source data 3.jpg]

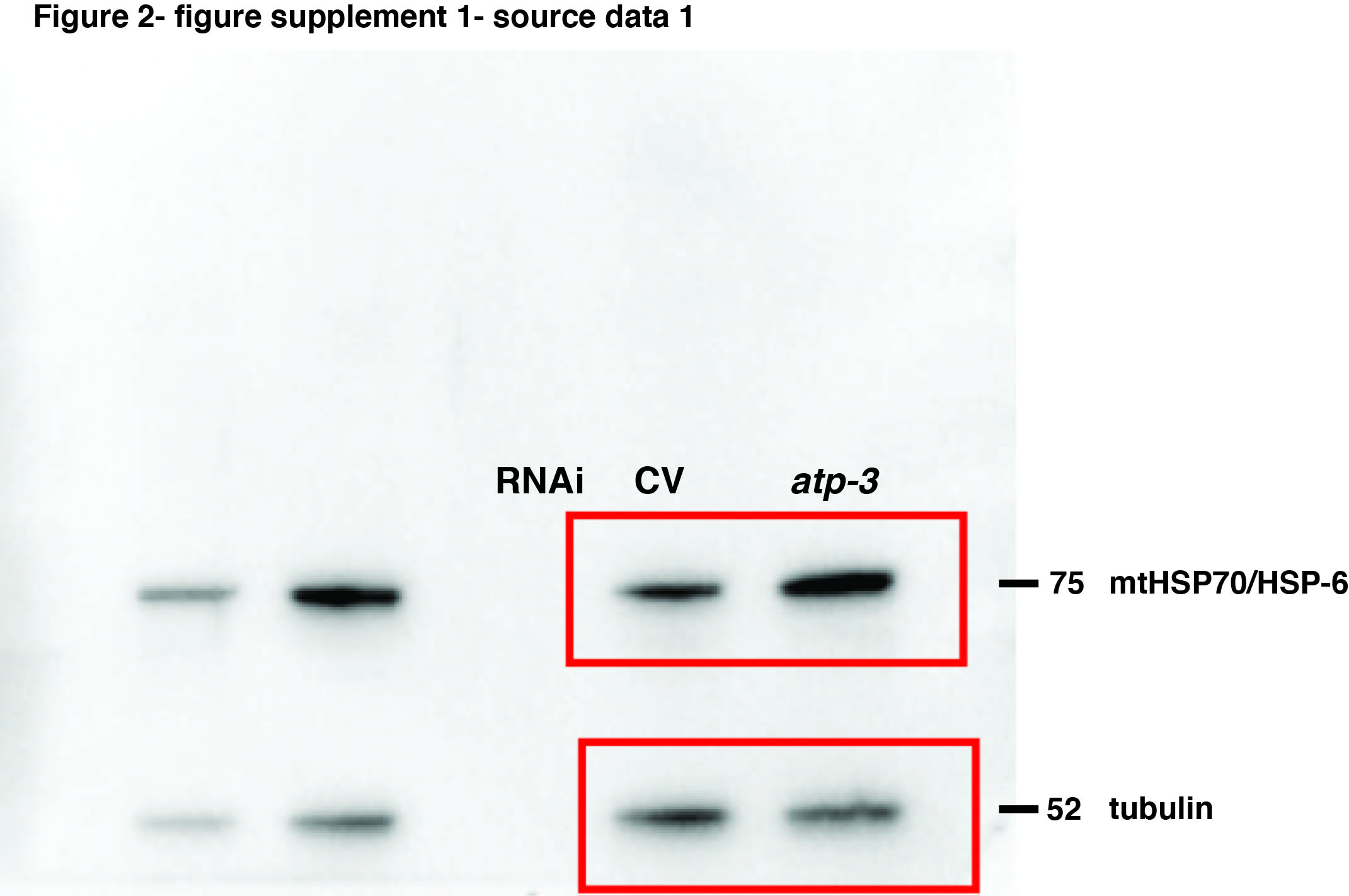

Supplement: Figure 2—figure supplement 1—source data 1. [file elife-63453-fig2-figsupp1-data1.jpg.zip › Figure 2- figure supplement 1- source data 1.jpg]

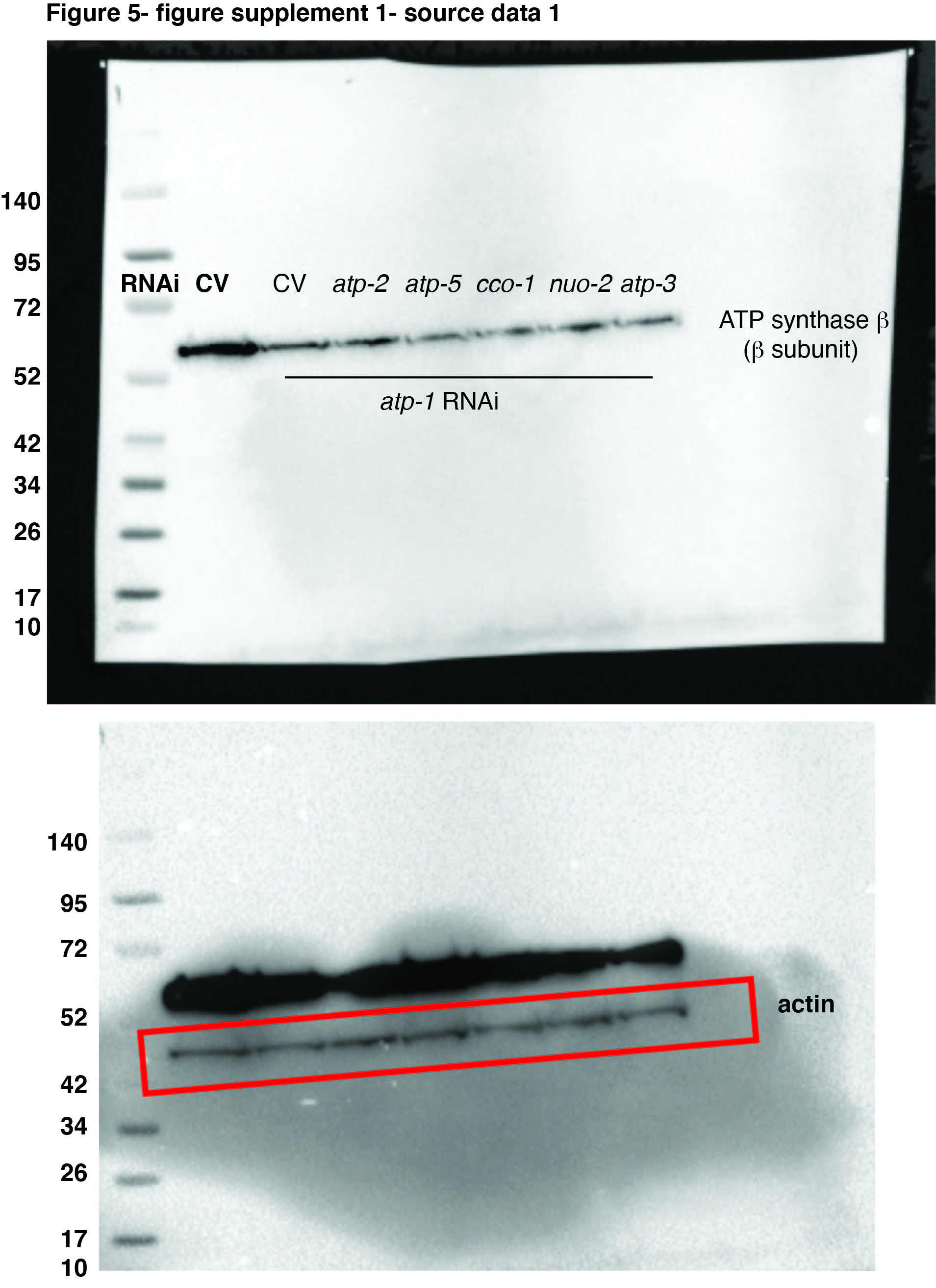

Supplement: Figure 5—figure supplement 1—source data 1. [file elife-63453-fig5-figsupp1-data1.jpg.zip › Figure 5- figure supplement 1- source data 1.jpg]
